# Evidence of Mott insulator with thermally induced melting behavior in kagome compound Nb_3_Cl_8_

**DOI:** 10.1093/nsr/nwaf464

**Published:** 2025-11-04

**Authors:** Qiu Yang, Min Wu, Jingyi Duan, Zhijie Ma, Lingxiao Li, Zihao Huo, Zaizhe Zhang, Kenji Watanabe, Takashi Taniguchi, Xiaoxu Zhao, Yi Chen, Youguo Shi, Wei Jiang, Kaihui Liu, Xiaobo Lu

**Affiliations:** International Center for Quantum Materials, School of Physics, Peking University, Beijing 100871, China; International Center for Quantum Materials, School of Physics, Peking University, Beijing 100871, China; Centre for Quantum Physics, Key Laboratory of Advanced Optoelectronic Quantum Architecture and Measurement (MOE), School of Physics, Beijing Institute of Technology, Beijing 100081, China; Beijing Key Lab of Nanophotonics & Ultrafine Optoelectronic Systems, School of Physics, Beijing Institute of Technology, Beijing 100081, China; Beijing National Laboratory for Condensed Matter Physics, Institute of Physics, Chinese Academy of Sciences, Beijing 100190, China; International Center for Quantum Materials, School of Physics, Peking University, Beijing 100871, China; International Center for Quantum Materials, School of Physics, Peking University, Beijing 100871, China; International Center for Quantum Materials, School of Physics, Peking University, Beijing 100871, China; Research Center for Electronic and Optical Materials, National Institute of Material Sciences, Tsukuba 305-0044, Japan; Research Center for Materials Nanoarchitectonics, National Institute of Material Sciences, Tsukuba 305-0044, Japan; School of Materials Science and Engineering, Peking University, Beijing 100871, China; International Center for Quantum Materials, School of Physics, Peking University, Beijing 100871, China; Beijing National Laboratory for Condensed Matter Physics, Institute of Physics, Chinese Academy of Sciences, Beijing 100190, China; Centre for Quantum Physics, Key Laboratory of Advanced Optoelectronic Quantum Architecture and Measurement (MOE), School of Physics, Beijing Institute of Technology, Beijing 100081, China; Beijing Key Lab of Nanophotonics & Ultrafine Optoelectronic Systems, School of Physics, Beijing Institute of Technology, Beijing 100081, China; State Key Laboratory for Mesoscopic Physics, Frontiers Science Centre for Nano-optoelectronics, School of Physics, Peking University, Beijing 100871, China; International Center for Quantum Materials, School of Physics, Peking University, Beijing 100871, China; Collaborative Innovation Center of Quantum Matter, Beijing 100871, China

**Keywords:** kagome lattice, flat band, Mott insulator

## Abstract

The kagome lattice provides a playground to explore novel correlated quantum states due to the presence of flat bands in its electronic structure. The recently discovered layered kagome compound Nb_3_Cl_8_ has been proposed as a Mott insulator coming from the half-filled flat band. Here, we have carried out a systematic transport study to uncover evidence of the Mott insulator in Nb_3_Cl_8_ thin flakes. A bipolar semiconducting property with Fermi level close to the conduction band has been revealed. We have further probed the chemical potential of Nb_3_Cl_8_ by tracing the charge neutrality point of monolayer graphene proximate to Nb_3_Cl_8_. The gap of Nb_3_Cl_8_ flakes is ∼1.10 eV at 100 K and shows pronounced temperature dependence, decreasing substantially with increasing temperature to ∼0.63 eV at 300 K. The melting behavior of the gapped state is consistent with the theoretically proposed Mott insulator in Nb_3_Cl_8_. Our work has demonstrated Nb_3_Cl_8_ as a promising platform to study strongly correlated physics at relatively high temperature.

## Teaser

The layered kagome material Nb_3_Cl_8_ exhibits a room-temperature Mott insulating state, highlighting its potential as a model system for studying strong electron correlations.

## INTRODUCTION

Electron correlation-induced Mott insulators serve as parent phases for numerous strongly correlated phenomena, including high-temperature superconductors and magnetism [1,2]. Mott insulators are commonly observed in materials such as layered perovskites (e.g. La_2_CuO_4_ [3–5], Sr_2_IrO_4_ [6–8]), monometallic oxides (e.g. V_2_O_3_ [9,10], NiO [11–13]), transition-metal dichalcogenides (e.g. NiS_2_ [14,15], 1T-TaS_2_ [16–18]) and so on. These systems exhibit complex many-body ground states that cannot be explained by the conventional band theory of solids. A hallmark feature of Mott insulators is the half-filled electronic band structure near the Fermi level, where the strong on-site Coulomb interactions localize the electrons, preventing charge transport and opening a Mott gap [19–21]. However, achieving precise control over the electronic state in conventional Mott insulators often relies on extreme conditions (such as high pressure, cryogenic temperatures) or irreversible methods such as chemical doping, thereby limiting the potential application of Mott materials.

The system exhibiting flat band offers a promising route toward realizing Mott insulating states, in which Coulomb interactions are significantly stronger than the kinetic energy, readily enabling strong electron correlations, such as in artificial superlattices [22–24]. Alternatively, flat band structures can be naturally engineered in one promising route: the kagome lattice [25–27]. The kagome lattice is a geometrically frustrated structure constituted by corner-sharing triangles, with the coexistence of Dirac cone, van Hove singularity and flat bands in its electronic structure, making this system a fertile platform on which to investigate the various quantum phenomena originating from the interplay between topology, geometry and correlation [28–30]. However, the correlated phenomena associated with the flat bands remain largely underexplored. One prime reason is that the flat bands in the metallic kagome materials lie away from the Fermi surface and intertwine with other bands [25,31–34], which precludes the exploration of correlated behavior contributed from the flat bands. Additionally, both the difficulty in producing ultra-thin flakes [35] (although the AV_3_Sb_5_ family is a van der Waals material [36]) and the absence of band gap in kagome metals limit their potential application in nano-electronic devices. Thus, layered semiconducting kagome materials with isolated flat bands near the Fermi level are exceptionally interesting.

**Figure 1.**
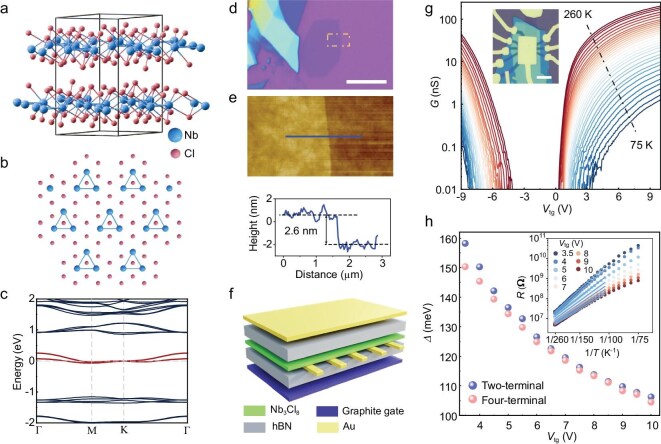
Fundamental properties of Nb_3_Cl_8_. (a, b) Crystal structure of Nb_3_Cl_8_. The Nb atoms in each layer form a trigonally distorted kagome lattice. (c) Non-interacting band structure of bulk Nb_3_Cl_8_ with a bilayer stacking periodicity. (d, e) Optical image of the exfoliated thin flake of Nb_3_Cl_8_. Scale bar: $10{\mathrm{\ \mu m}}$. (e) Atomic force microscopy image of Nb_3_Cl_8_ and the height profile along the the blue line. The corresponding thickness is ∼2.6 nm measured by using atomic force microscopy. (f) Schematic of hBN-encapsulated dual-gated device of Nb_3_Cl_8_. The top gate and electrodes are made of Au. (g) Two-terminal conductance of Nb_3_Cl_8_ as a function of the top gate voltage. Insert: optical image of the device S1, scale bar: $10{\mathrm{\ \mu m}}$. (h) Thermal activation gap measured with two-terminal (blue dots) and four-terminal (pink dots) configuration as a function of the top gate voltage. Insert shows the experimental data from 260 to 100 K fitted by using the Arrhenius formula. During the measurements of device S1, the applied bottom gate voltage is equal to the top gate voltage.

Recently, a new family of van der Waals kagome compounds, Nb_3_*X*_8_ (*X* = Cl, Br, I), has been proposed as an ideal system in which to study the strongly correlated physics [37–43]. In these layered materials, the Nb atoms in each layer form a trigonally distorted kagome lattice, namely breathing kagome lattice, as shown in Fig. 1a and b for Nb_3_Cl_8_. Angle-resolved photoemission spectroscopy (ARPES) experiments with calculations of Nb_3_Cl_8_ crystals have revealed that the flat band is separated from other bands, away from the Fermi level, and a single-particle band gap arising from symmetry breaking opened at the Fermi surface [38]. However, the latest ARPES measurements and calculations present different results—the flat band lies near the Fermi level and is half-filled, giving rise to a Mott gap that can be described by using single-band Hubbard model [40]. Understanding the nature of the ground state is very instructive for further exploring the fascinating electronic phases in Nb_3_Cl_8_.

In this work, we fabricated hexagonal boron nitride (hBN)-encapsulated dual-gated devices of Nb_3_Cl_8_ thin flakes. This allows us to directly measure the resistance modulated by the gate voltages and assess its relationship with temperature. Electronic transport measurements reveal that Nb_3_Cl_8_ exhibits semiconducting behavior with ambipolar characteristics. To investigate the nature of the band gap, monolayer graphene (MLG) was employed to monitor the chemical potential $\mu $ of Nb_3_Cl_8_. A gap of ∼1.10 eV was observed at 100 K via tracking the charge neutrality point (CNP) of MLG, exhibiting significant temperature dependence with rapid reduction at elevated temperatures. This behavior strongly contrasts with that of the conventional single-particle gap, in which the gap remains nearly temperature-independent, and can be attributed to the emergence of a Mott gap driven by strong electron correlations.

## RESULTS

Figure 1c shows the non-interacting band structure of bulk Nb_3_Cl_8_ with a bilayer stacking periodicity, in which flat bands cross the Fermi level in individual layers with half-filled occupation states. Notably, the number of half-filled flat bands at the Fermi surface in the single-particle framework equals the stacking periodicity in the bulk Nb_3_Cl_8_. Due to the exceptionally weak coupling between neighboring layers, the electronic properties of each layer are primarily dominated by intralayer characteristics. The flat band in monolayer Nb_3_Cl_8_ is also half-filled across the Fermi level without electron correlations (as shown in Fig. S4). Nb_3_Cl_8_ thin flakes can be readily mechanically exfoliated from the bulk crystal; Fig. 1d shows the optical image of a typical Nb_3_Cl_8_ thin flake with a thickness of ∼2.6 nm, determined by using atomic force microscopy (Fig. 1e). The hBN-encapsulated dual-gated device, as schematically illustrated in Fig. 1f, was fabricated by using dry transfer techniques and standard nanofabrication techniques. The two-terminal conductance of a typical 2.6-nm-thick sample S1 (inset in Fig. 1g), as a function of the gate voltages, exhibits a bipolar semiconducting characteristic that is slightly electron-doped, as shown in Fig. 1g. Similar behavior has been observed in device S2, which has a thickness of 4.2 nm (see Fig. S3).

Interestingly, even when highly doped with electrons, i.e., at ${V}_{{\mathrm{tg}}} = \ {V}_{{\mathrm{bg}}} = \ 10\ {\mathrm{V}}$, corresponding to a carrier density of ∼$n\ = \ 1.3 \times {10}^{13}\ {\mathrm{c}}{{\mathrm{m}}}^{ - 2}$, the sample exhibits robust insulating behavior with clear thermal activation observed over a wide temperature range (100–260 K). By using the Arrhenius formula $R{\mathrm{\ }} \propto {\mathrm{\ exp\ [- }}\Delta {\mathrm{/2}}{k}_{\mathrm{B}}T{\mathrm{]}}$, where ${k}_{\mathrm{B}}$ is the Boltzmann constant, the thermal activation gap $\Delta $ as a function of ${V}_{{\mathrm{tg}}}$ is quantitatively depicted in Fig. 1h, with the insert showing the two-terminal resistance versus temperature at different ${V}_{{\mathrm{tg}}}$. We note that the four-terminal measurements (shown in Fig. S2) illustrate essentially the same features, including the magnitude and variation of $\Delta $ with ${V}_{{\mathrm{tg}}}$, as marked by the pink dots in Fig. 1h. Therefore, we consider the two-terminal data presented here to be compelling and they provide a definitive representation of the fundamental properties of Nb_3_Cl_8_ flakes.

Surprisingly, a gap of up to ∼105 meV can still be revealed at ${V}_{{\mathrm{tg}}} = \ {V}_{{\mathrm{bg}}} = \ 10\ {\mathrm{V}}$, highly indicating the presence of strong electron interactions. The interaction strength can be qualitatively described by using the ratio between the potential energy and the kinetic energy, which can be expressed as ${r}_{\mathrm{s}}{\mathrm{\, =\, }}\frac{{{n}_{\mathrm{\nu }}{m}^{\mathrm{*}}{{\mathrm{e}}}^{\mathrm{2}}}}{{{\mathrm{4\pi }}\varepsilon {\hbar }^{\mathrm{2}}\sqrt {{\mathrm{\pi }}n} }}$, where ${n}_\nu $ is the number of degenerate valleys, $\varepsilon $ is the dielectric constant and ${m}^*$ is the effective electron mass [44]. For Nb_3_Cl_8_, we calculated ${r}_{\mathrm{s}} \approx 24.8\ ( {60.9} )\ $for the valence (conduction) band, with ${n}_\nu = 1$,$\ {m}^* = - 2.53{m}_0\ ( {6.20{m}_0} )$, $\varepsilon \ = \ 3{\varepsilon }_0\ $(the effective electron mass and dielectric constant are calculated from the band structure of Nb_3_Cl_8_ and the geometric capacitance of the MLG/hBN/Nb_3_Cl_8_ system, respectively, as described in the Supplementary information), and $n\ = \ 1.3 \times {10}^{13}{\mathrm{\ c}}{{\mathrm{m}}}^{ - 2}$ for the largest carrier density in our experiments. The ultra-high ${r}_{\mathrm{s}}$ value confirms that Nb_3_Cl_8_ is a strongly correlated system, potentially giving rise to exotic phenomena such as the existence of an intrinsic Wigner crystal.

**Figure 2.**
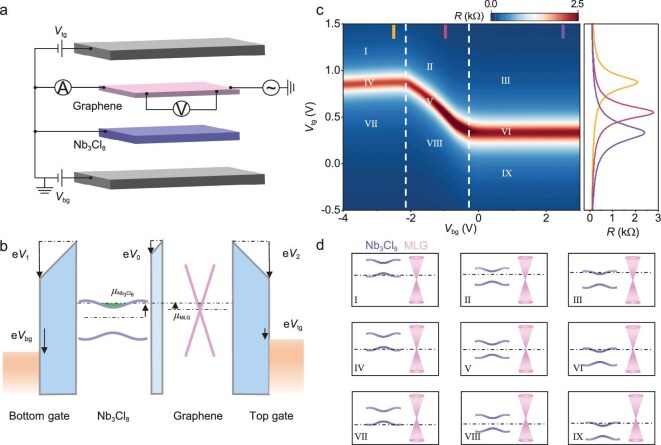
MLG/hBN/Nb_3_Cl_8_ heterostructure device. (a) Schematic of the methodology. MLG and Nb_3_Cl_8_ are separated by a thin hBN layer. (b) Band alignment of MLG and Nb_3_Cl_8_, illustrating the relationship between the chemical potentials of MLG (${\mu }_{{\mathrm{MLG}}}$) and Nb_3_Cl_8_ (${\mu }_{{\mathrm{Nb_3Cl_8}}}$), with the control of the top gate voltage (${V}_{{\mathrm{tg}}}$), bottom gate voltage (${V}_{{\mathrm{bg}}}$) and the electrostatic potential drops ${V}_0$, ${V}_1$, ${V}_2$. *e* is the elementary charge. (c) Resistance of MLG measured at 200 K. This 2D color map can be divided into nine regions based on the trace of the CNP and its inflection points (indicated by the dashed white lines) of the MLG. The line cuts of MLG resistance are shown on the right panel of (c). (d) Band alignments of MLG and Nb_3_Cl_8_ across the nine regions shown in (c).

Furthermore, to investigate the nature of the gap, MLG was employed to probe the chemical potential $\mu $ of Nb_3_Cl_8_, as shown in the schematic diagram of the measurement configuration in Fig. 2a. Such a methodology has been widely used to track the chemical potential in various 2D systems [45–48]. In the heterostructure devices, MLG and Nb_3_Cl_8_ thin flakes are separated by a thin hBN layer (∼5 nm). Figure 2b shows the band alignment of MLG and Nb_3_Cl_8_ with the control of double gates. Here, we set ${\mu }_{{\mathrm{MLG}}} = 0$ when the carrier density of MLG, ${n}_{{\mathrm{MLG}}}{\mathrm{\ = \ 0}}$, corresponding to the CNP. Then, the chemical potential ${\mu }_{{\mathrm{Nb_3Cl_8}}}$ and carrier density ${n}_{{\mathrm{N}}{{\mathrm{b}}}_3{\mathrm{C}}{{\mathrm{l}}}_8}$ of the Nb_3_Cl_8_ flake are given by:


(1) \begin{eqnarray*}
{\mu }_{{\mathrm{N}}{{\mathrm{b}}}_3{\mathrm{C}}{{\mathrm{l}}}_8} = - \frac{{e{C}_{{\mathrm{tg}}}{V}_{{\mathrm{tg}}}}}{{{C}_{{\mathrm{eff}}}}},
\end{eqnarray*}

(2) \begin{eqnarray*}
{n}_{{\mathrm{N}}{{\mathrm{b}}}_3{\mathrm{C}}{{\mathrm{l}}}_8} = \frac{{{C}_{{\mathrm{bg}}}{V}_{{\mathrm{bg}}}}}{e} + \frac{{\left( {{C}_{{\mathrm{bg}}} + {C}_{{\mathrm{eff}}}} \right){C}_{{\mathrm{tg}}}{V}_{{\mathrm{tg}}}}}{{e{C}_{{\mathrm{eff}}}}},
\end{eqnarray*}

where ${C}_{{\mathrm{eff}}}\ $is the effective geometric capacitances per unit area considering both the dielectric properties of hBN and Nb_3_Cl_8_, *e* is the elementary charge, and ${C}_{{\mathrm{tg}}}$ and ${C}_{{\mathrm{bg}}}$ are the geometric capacitances per unit area of the top hBN and bottom hBN, respectively (see Supplementary information for detailed derivation). Apparently, the evolution of the CNP in MLG can reflect the carrier density ${n}_{{\mathrm{Nb_3Cl_8}}}$-dependent chemical potential ${\mu }_{{\mathrm{Nb_3Cl_8}}}\ $of the Nb_3_Cl_8_ flake.

Figure 2c presents the four-terminal resistance of MLG as a function of ${V}_{{\mathrm{tg}}}$ and ${V}_{{\mathrm{bg}}}$ at *T* = 200 K. Based on carrier doping, the 2D color map can be divided into nine regions, as shown in Fig. 2c, and the corresponding band alignments between MLG and Nb_3_Cl_8_ are depicted in Fig. 2d. The doping states of Nb_3_Cl_8_ are divided into three distinct regions: hole-doped, charge-neutral and electron-doped (from left to right), based on the two white dashed lines at ${V}_{{\mathrm{bg}}} = - 2.11\ {\mathrm{V}}$ and $- 0.35\ {\mathrm{V}}$. The resistance at the Dirac point of MLG varies, depending on the type of carrier doped into the Nb_3_Cl_8_, as shown on the right side of Fig. 2c. Owing to the screening effect from the MLG, the Fermi level of the Nb_3_Cl_8_ is slightly dependent on the top gate ${V}_{{\mathrm{tg}}}$. For MLG, the CNP is characterized by a resistance peak, which clearly defines the transition between the electron-doping and hole-doping regions. Noted that the track of the CNP does not pass through the point at ${V}_{{\mathrm{tg}}} = 0$ and ${V}_{{\mathrm{bg}}} = 0$, which can be attributed to the type-Ⅲ band alignment between MLG and Nb_3_Cl_8_, as illustrated in Fig. 2d-Ⅸ. When MLG was placed closed to Nb_3_Cl_8_, the electrons in the MLG spontaneously transferred to the Nb_3_Cl_8_, even though these two thin flakes were isolated by the spacer layer of hBN, similarly to the graphene/hBN/RuCl_3_ device previously reported [49]. In this case, the Fermi level for MLG should be positioned in the valence band instead of the CNP at the initial state (${V}_{{\mathrm{tg}}} = 0$, ${V}_{{\mathrm{bg}}} = 0$) due to the charge-transfer behavior.

**Figure 3.**
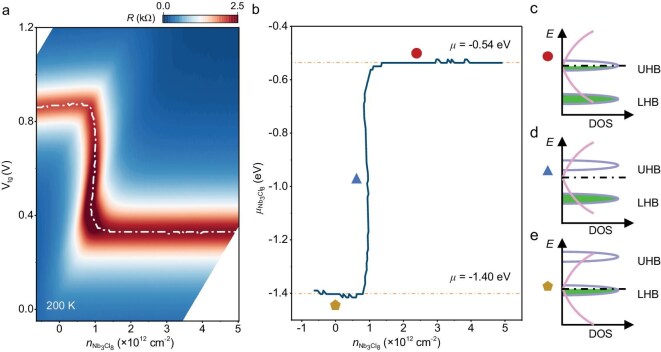
Chemical potential of Nb_3_Cl_8_. (a) Resistance of MLG versus ${V}_{{\mathrm{tg}}}$ and the carrier density of Nb_3_Cl_8_ at 200 K. The white lines indicate the CNP positions of the graphene. By tracking the position of the CNP, where all charge carriers reside in Nb_3_Cl_8_ layers, the relative chemical potential of Nb_3_Cl_8_ (linear with ${V}_{{\mathrm{tg}}}$) as a function of ${n}_{{\mathrm{Nb_3Cl_8}}}$ can be obtained. (b) Chemical potential of Nb_3_Cl_8_ versus its carrier density, extracted from the map in (a). The chemical potential of Nb_3_Cl_8_ is pinned at $- 1.40{\mathrm{\ eV}}$ on the hole side and $- 0.54{\mathrm{\ eV}}$ on the electron side, yielding a gap with a value equal to $\Delta {\mu }_{{\mathrm{Nb_3Cl_8}}} \sim 0.86{\mathrm{\ eV}}$. (c–e) Hubbard band filling of Nb_3_Cl_8_. The Fermi level remains at the lower Hubbard band (e) when the carrier density is <$1 \times {10}^{12}\ {\mathrm{c}}{{\mathrm{m}}}^{ - 2}$; as the carrier density increases, the Fermi level rises to the Mott gap (d) and eventually reaches the upper Hubbard band (c).

**Figure 4.**
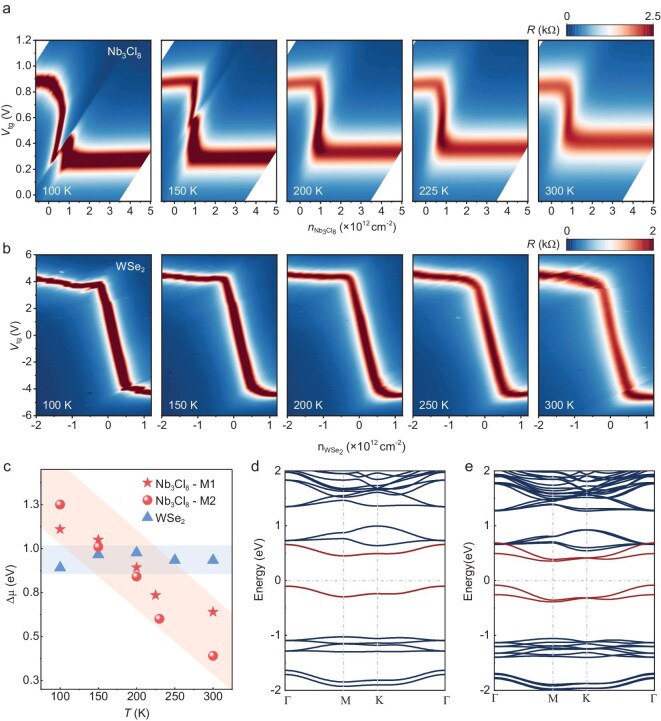
Mott insulating behavior of Nb_3_Cl_8_. (a, b) Four-terminal resistance of MLG measured from $100{\mathrm{\ }}$to $300{\mathrm{\ K}}$ on the device of (a) MLG/hBN/Nb_3_Cl_8_ and (b) MLG/hBN/WSe_2_ heterostructures, respectively. The chemical potential of the Nb_3_Cl_8_/bilayer WSe_2_ can be obtained according to the CNP of MLG. (c) Gap of Nb_3_Cl_8_ and bilayer WSe_2_ from $100\ $to $300\ {\mathrm{K}}$, calculated based on the difference in chemical potential between the electron- or hole-doped regime. As the temperature increases, the gap of Nb_3_Cl_8_ (represented by stars and dots for devices M1 and M2, respectively) decreases sharply, in strong contrast to that of WSe_2_ (triangles), which is nearly independent of temperature. (d, e) Band structure of (d) monolayer and (e) bulk Nb_3_Cl_8_ after considering the electron correlation effect with Hubbard term ${U}_{{\mathrm{eff}}} = 2{\mathrm{\ eV}}$. The strong electron correlations split the half-filled band near the Fermi level (shown in Fig. 1c and Fig. S4a) into upper and lower Hubbard bands, giving rise to a Mott gap.

The phase diagram of MLG resistance versus the gate voltage (Fig. 2c) can be transformed into the parameter space of ${V}_{{\mathrm{tg}}}- {n}_{{\mathrm{Nb_3Cl_8}}}$ by using Equations (1) and (2), as plotted in Fig. 3a, which conveniently illustrates the evolution of the chemical potential ${\mu }_{{\mathrm{Nb_3Cl_8}}}$ with continuous hole or electron doping at *T* = 200 K. The chemical potential of Nb_3_Cl_8_ (${\mu }_{{\mathrm{Nb_3Cl_8}}}$) can be determined by tracking the CNP position of MLG and ${\mu }_{{\mathrm{Nb_3Cl_8}}}$ exhibits a linear dependence on the ${V}_{{\mathrm{tg}}}$ applied to the CNP region, as theoretically illustrated by Equation (1). Figure 3b shows the variation in the chemical potential ${\mu }_{{\mathrm{Nb_3Cl_8}}}\ $as a function of ${n}_{{\mathrm{Nb_3Cl_8}}}$ by tracking the CNP of MLG (solid white line in Fig. 3a). On the electron side, ${\mu }_{{\mathrm{Nb_3Cl_8}}}$ saturates at approximately –0.54 eV. As the carrier density decreases, ${\mu }_{{\mathrm{Nb_3Cl_8}}}$ sharply decreases and approaches –1.40 eV on the hole side, whereupon, the magnitude of gap ${\mathrm{\Delta }}\mu $ in the Nb_3_Cl_8_ is ∼0.86 eV at 200 K, which is consistent with the theoretical calculations and the optical experiments [38,40]. The ${\mu }_{{\mathrm{Nb_3Cl_8}}}$ is almost a constant (Figs 3 and 4a) whether on the electron or hole side, which implies the existence of flat bands near the Fermi level in Nb_3_Cl_8_. It is noted that the jump in ${\mu }_{{\mathrm{Nb_3Cl_8}}}$ occurs at ∼${n}_{{\mathrm{Nb_3Cl_8}}} = 1 \times {10}^{12}{\mathrm{c}}{{\mathrm{m}}}^{ - 2}$, rather than ${n}_{{\mathrm{Nb_3Cl_8}}} = 0$, and the extracted ${\mu }_{{\mathrm{Nb_3Cl_8}}}$ is always negative, which further demonstrates the charge-transfer doping in the MLG/hBN/Nb_3_Cl_8_ heterostructure.

Figure 4a displays the temperature-dependent phase diagram of MLG measured from 100 to 300 K, with the corresponding ${\mathrm{\Delta }}{\mu }_{{\mathrm{Nb_3Cl_8}}}$ plotted in Fig. 4c, marked by stars. Obviously, the magnitude of gap ${\mathrm{\Delta }}{\mu }_{{\mathrm{Nb_3Cl_8}}}$ is ∼1.10 eV at 100 K and is then reduced to 0.63 eV at 300 K. Similar results were also observed in device M2, as marked with dots. This dramatic decreasing phenomenon with increasing temperature is in strong contrast to the characteristics of conventional semiconductors, in which the band gaps change only slightly with increasing temperature [50,51]. To further confirm the difference between Nb_3_Cl_8_ and conventional semiconductors, we probed the chemical potential $\mu $ of bilayer WSe_2_, the band gap of which is ∼1.2 eV [52,53]. Similarly to the structure of the MLG/hBN/Nb_3_Cl_8_ heterostructure device, bilayer WSe_2_ and MLG are separated by a thin hBN layer. Figure 4b shows the four-terminal resistance of MLG on the parameter space of ${V}_{{\mathrm{tg}}}$–${n}_{{\mathrm{WSe_2}}}$ from 100 to 300 K on the device MLG/hBN/WSe_2_ heterostructure. Clearly, the CNP of MLG crosses ${V}_{{\mathrm{tg}}} = 0$ and ${n}_{{\mathrm{WSe_2}}} = 0$, in contrast to that observed in Figs 2c and 3a, which is accordance with the band alignment between MLG and WSe_2_ [54]. Similarly, the chemical potential of WSe_2_ can be obtained by the CNP of MLG. As shown in Fig. 4c, marked with triangles, the band gap in bilayer WSe_2_ is ∼1 eV and nearly unchanged with temperature.

The evolution of the band gap in Nb_3_Cl_8_ with temperature is reminiscent of strong electron interactions generated Mott insulator, in which the rate of band-gap reduction is significantly faster than that in conventional semiconductors. For the latter, the band gap is primarily governed by the static electronic structure, with minimal temperature dependence. However, the Mott gap is highly temperature-dependent due to the strong electron correlations. At elevated temperatures, thermal fluctuations may disrupt the strong electron correlations, thereby facilitating the previously suppressed electronic transitions and leading to a significant reduction in the Mott gap, with the decrease in the gap size being an order of magnitude larger than ${k}_{\mathrm{B}}T\ $[55,56].

Figure 4d and e show the band structures for both monolayer and bulk Nb_3_Cl_8_ based on density functional theory plus Hubbard U correction (DFT + U), respectively. With the electron correlations having been considered, the half-filled flat band near the Fermi level splits into upper and lower Hubbard bands, giving rise to a Mott gap. Similar to the band structure in Fig. 1c, the number of upper and lower Hubbard bands in the single-particle framework equals the stacking periodicity in bulk Nb_3_Cl_8_. When the chemical potential of Nb_3_Cl_8_ is pinned at –1.40 eV, as shown in Fig. 3b, the Fermi level remains at the lower Hubbard band, as shown in Fig. 3e. As the carrier density increases, the Fermi level crosses the gap and eventually reaches the upper Hubbard band, as shown in Fig. 3d and c. Noted that Fig. 3c–e serve as an effective schematic to illustrate the evolution of the electronic structure in the low-doping regime. When the doping level becomes higher, the Fermi level will not simply enter the lower or upper Hubbard bands. Instead, the electronic structure will reshuffle, whereupon the observed insulating behavior at high temperatures, significant temperature-dependent band-gap reduction and band-structure calculations collectively provide compelling evidence of Mott insulating behavior in Nb_3_Cl_8_, which persists up to room temperature. Furthermore, the Mott state is related to the localization of one electron on each Nb_3_ cluster, which yields a [Nb_3_]^8+^ valence state with localized S = 1/2 moments [57]. This cluster magnetism makes Nb_3_Cl_8_ a promising platform for exploring exotic magnetic phases, such as quantum spin liquids.

## DISCUSSION

In summary, we performed systematic electronic transport measurements to reveal the evidence of Mott states in Nb_3_Cl_8_. The bipolar semiconducting characteristic was observed by using direct measurements on Nb_3_Cl_8_ flakes. By employing MLG as the detector layer, the gap size of the Nb_3_Cl_8_ flakes, extracted from the chemical potential difference between the hole- and electron-doped regions, is highly sensitive to temperature. This behavior strongly contrasts with that of conventional semiconductors and is attributed to the formation of the Mott gap. The room-temperature Mott insulating behavior in Nb_3_Cl_8_ provides a promising platform for investigating strongly correlated physics as well as moiré engineering in the future.

## Supplementary Material

nwaf464_Supplemental_File
