# Supplementary material for: Evidence of Mott insulator with thermally induced melting behavior in kagome compound Nb3Cl8
Source: Natl Sci Rev. 2025 Nov 4;12(12):nwaf464. doi: 10.1093/nsr/nwaf464 (PMC12781896; doi:10.1093/nsr/nwaf464)
Supplement: nwaf464_Supplemental_File [file nwaf464_supplemental_file.pdf]

Supporting Information for

Evidence of Mott Insulator with Thermally Induced Melting Behavior in Kagome Compound Nb_3Cl_8

Qiu Yang[1,†], Min Wu[1,†,*], Jingyi Duan[2,3,†], Zhijie Ma[4], Lingxiao Li[1], Zihao Huo[1], Zaizhe Zhang[1], Kenji Watanabe[5], Takashi Taniguchi[6], Xiaoxu Zhao[7], Yi Chen[1], Youguo Shi[4], Wei Jiang[2,3], Kaihui Liu[8,*], Xiaobo Lu[1,9,*]

[1]International Center for Quantum Materials, School of Physics, Peking University, Beijing 100871, China
[2]Centre for Quantum Physics, Key Laboratory of Advanced Optoelectronic Quantum Architecture and Measurement (MOE), School of Physics, Beijing Institute of Technology, Beijing 100081, China
[3]Beijing Key Lab of Nanophotonics & Ultrafine Optoelectronic Systems, School of Physics, Beijing Institute of Technology, Beijing 100081, China
[4]Beijing National Laboratory for Condensed Matter Physics, Institute of Physics, Chinese Academy of Sciences, Beijing 100190, China
[5]Research Center for Electronic and Optical Materials, National Institute of Material Sciences, 1-1 Namiki, Tsukuba 305-0044, Japan
[6]Research Center for Materials Nanoarchitectonics, National Institute of Material Sciences, 1-1 Namiki, Tsukuba 305-0044, Japan
[7]School of Materials Science and Engineering, Peking University, Beijing, 100871, China
[8]State Key Laboratory for Mesoscopic Physics, Frontiers Science Centre for Nano-optoelectronics, School of Physics, Peking University, Beijing 100871, China
[9]Collaborative Innovation Center of Quantum Matter, Beijing 100871, China

[†] Equally contributed to this work.
[*]Corresponding authors. Emails: min.wu@pku.edu.cn, khliu@pku.edu.cn, xiaobolu@pku.edu.cn

Device Fabrication

The devices were fabricated by a layer-by-layer dry transfer method with a poly (bisphenol A carbonate)/polydimethylsiloxane (PC/PDMS) stamp [1]. Thin flakes, including monolayer graphene, few-layer graphite, Nb_3Cl_8, and hexagonal boron nitride (hBN) were mechanically exfoliated onto 285 nm SiO_2/Si substrates from bulk crystals. The thickness of Nb_3Cl_8 and hBN were measured using atomic force microscope (AFM). We fabricated two kinds of devices: S1, S2 and M1, M2.

For devices S1 and S2, the hBN/graphite stack was released onto a silicon substrate serve as the bottom gate. A Hall bar configuration was patterned on the hBN dielectric layer using standard e-beam lithography, followed by thermal evaporation of Ti (2 nm)/Au (8 nm) electrodes and lift-off processing. The electrodes were annealed at 350 ℃ for 4 hours to remove polymer and resist residues, followed by cleaning with AFM in contact mode to ensure optimal electrical contact. The top hBN/Nb_3Cl_8 stack was assembled through layer-by-layer transfer and released onto the pre-prepared Ti/Au electrodes. Finally, a metallic top gate Cr (5 nm)/ Au (60 nm) was deposited via standard e-beam lithography and thermal evaporation.

The devices M1 and M2 consist of monolayer graphene (MLG) and Nb_3Cl_8, separated by a thin hBN layer (~5 nm). The layer transfer sequence as following: graphite top gate, top hBN, MLG, spacer hBN, Nb_3Cl_8, contact graphite electrodes, bottom hBN, and graphite bottom gate. The Hall bar geometry for the transport measurements was patterned using e-beam lithography and reactive ion etching. Finally, Cr (5 nm)/Au (60 nm) electrodes was deposited via standard e-beam lithography and thermal evaporation.

Chemical potential measurement

As the top layer in the double-layer heterostructure, graphene serves as an effective sensor for probing the chemical potential of the target bottom layer [2–5]. As shown in Fig. 2b of main text, we assumed $V_{0,1,2}$ denotes the electric potential variation across the dielectric layer of intermediate/bottom/top gates. When the system is in equilibrium, we have the following relationships [4,5]:

$$eV_{\mathrm{bg}} = eV_1 + \mu_{\mathrm{Nb_3Cl_8}}\left(n_{\mathrm{Nb_3Cl_8}}\right)$$

$$eV_{\mathrm{tg}} = eV_2 + \mu_{\mathrm{MLG}}(n_{\mathrm{MLG}})$$

$$eV_0 = \mu_{\mathrm{Nb_3Cl_8}}\left(n_{\mathrm{Nb_3Cl_8}}\right) - \mu_{\mathrm{MLG}}(n_{\mathrm{MLG}})$$

where, $\mu_{\mathrm{Nb_3Cl_8}}$ (μ_{MLG}) and $n_{\mathrm{Nb_3Cl_8}}$ (n_{MLG}) are the chemical potential and carrier density of Nb_3Cl_8 (MLG), respectively. e is the elementary charge, $V_{\mathrm{bg}}/V_{\mathrm{tg}}$ denotes the bottom/top gate voltage.

The carrier densities $n_{\mathrm{Nb_3Cl_8}}$ and n_{MLG} satisfy the electrostatic equations:

$$en_{\mathrm{Nb_3Cl_8}} = C_{\mathrm{bg}}V_1 - C_{\mathrm{i}}V_0$$
$$en_{\mathrm{MLG}} = C_{\mathrm{tg}}V_2 + C_{\mathrm{i}}V_0$$

where C_{bg}, C_{tg} and C_{i} are the geometric capacitances per unit area of the bottom, top and interlayer hBN dielectric layers, respectively. Considering the semiconducting property of Nb_3Cl_8, the geometric capacitance of C_{i} should include this component and be substituted by the effect geometric capacitance C_{eff}:

$$C_{\mathrm{eff}} = \frac{C_{\mathrm{i}} \cdot C_{\mathrm{Nb_3Cl_8}}}{C_{\mathrm{i}} + C_{\mathrm{Nb_3Cl_8}}}$$

where $C_{\mathrm{Nb_3Cl_8}}$ is the geometric capacitance per unit area of the Nb_3Cl_8, and we can calculate the dielectric constant $\varepsilon_{\mathrm{Nb_3Cl_8}}$ according to the geometric capacitance: $C_{\mathrm{Nb_3Cl_8}} = \frac{\varepsilon_0 \varepsilon_{\mathrm{Nb_3Cl_8}}}{d_{\mathrm{Nb_3Cl_8}}}$.

Combining the above six equations, we then obtained

$$en_{\mathrm{Nb_3Cl_8}} = C_{\mathrm{bg}}\left(V_{\mathrm{bg}} - \frac{\mu_{\mathrm{Nb_3Cl_8}}(n_{\mathrm{Nb_3Cl_8}})}{e}\right) + C_{\mathrm{eff}}\left(\frac{\mu_{\mathrm{MLG}}(\mathrm{n_{MLG}})}{\mathrm{e}} - \frac{\mu_{\mathrm{Nb_3Cl_8}}(\mathrm{n_{Nb_3Cl_8}})}{\mathrm{e}}\right)$$

$$en_{MLG} = C_{tg}\left(V_{tg} - \frac{\mu_{\mathrm{MLG}}(n_{\mathrm{MLG}})}{e}\right) - C_{\mathrm{eff}}\left(\frac{\mu_{\mathrm{MLG}}(\mathrm{n_{MLG}})}{\mathrm{e}} - \frac{\mu_{\mathrm{Nb_3Cl_8}}(\mathrm{n_{Nb_3Cl_8}})}{\mathrm{e}}\right)$$

At MLG charge neutrality point, where $n_{\mathrm{MLG}} = 0$ and $\mu_{\mathrm{MLG}} = 0$, the chemical potential and carrier density of Nb_3Cl_8 are given by

$$n_{\mathrm{Nb_3Cl_8}} = \frac{C_{\mathrm{bg}}V_{\mathrm{bg}}}{e} + \frac{(C_{\mathrm{bg}} + C_{\mathrm{eff}})C_{\mathrm{tg}}V_{\mathrm{tg}}}{eC_{\mathrm{eff}}}$$

$$\mu_{\mathrm{Nb_3Cl_8}} = -\frac{eC_{\mathrm{tg}}V_{\mathrm{tg}}}{C_{\mathrm{eff}}}$$

DFT calculation methods

The total-energy electronic-structure calculations were conducted utilizing first-principles methods rooted in Density Functional Theory (DFT). We employed the generalized gradient approximation exchange-correlation potentials, supplemented by the projector augmented wave [6] method for electron-ion interaction, as implemented in the Vienna Ab initio Simulation Package (VASP) code [7]. All self-consistent calculations were performed with a plane-wave cutoff energy of 500 eV. Geometric optimizations were executed without constraints until the force acting on each atom was less than 0.01 eV/Å, and the energy variation per cell was less than 10^{-5}eV. Information pertaining to the Brillouin zone k-points grid with a separation of $<$

$0.02\,\text{Å}^{-1}$. A more than $20\,\text{Å}$ vacuum space was included to avoid interactions between neighboring slabs. DFT+U method with on-site Hubbard U term was adopted to treat the correlation effect with multiple U values tested [8]. Interlayer van der Waals forces were also factored into the calculations, adopting a parameter setting of IVDW = 12 [9, 10].

Effective electronic mass calculations

Effective mass is defined based on the formula below:

$$m^* = \pm\hbar^2\left(\frac{d^2E_k}{dk^2}\right)^{-1}$$

The atomic unit system used by VASP is unified from the International System of Units, which need to be converted to electron mass m_0

$$m^* = 2.78029\times 10^{-29} J\cdot s^2/m^2\left(\frac{d^2E_k^{\text{vasp}}}{d(k^{\text{vasp}})^2}\right)^{-1}$$

$$m^* = 305.2m_0\left(\frac{d^2E_k^{\text{vasp}}}{d(k^{\text{vasp}})^2}\right)^{-1}$$

E_k^{vasp} *and* k^{vasp} are energy and momentum terms obtained from VASP ,

$$E_k^{\text{vasp}} = C + B_1k^{\text{vasp}} + B_2(k^{\text{vasp}})^2 + \cdots\cdots$$

$$\frac{d^2E_k^{\text{vasp}}}{d(k^{\text{vasp}})^2} = \frac{d^2C+B_1k^{\text{vasp}}+B_2(k^{\text{vasp}})^2+\cdots\cdots}{d(k^{\text{vasp}})^2} = 2B_2 + \cdots\cdots$$

based on which we are able to obtain the effective mass as,

$$m^* = 305.2m_0\left(\frac{1}{2B_2}\right)$$

According to Fig. S6, the fitted B_2 of the conduction band bottom and valence band top are equal to 24.62 and 60.36, respectively, and the corresponding effective masses are equal to $6.20m_0$ and $-2.53m_0$.

REFERENCES

1. Wang L, Meric I, Huang PY *et al.* One-dimensional electrical contact to a two-dimensional material. *Science* 2013; **342**: 614–7.

2. Kim S, Jo I, Nah J *et al.* Coulomb drag of massless fermions in graphene. *Phys Rev B* 2011; **83**: 161401.

3. Kim S, Jo I, Dillen DC *et al.* Direct measurement of the Fermi energy in graphene using a double-layer heterostructure. *Phys Rev Lett* 2012; **108**: 116404.

4. Park JM, Cao Y, Watanabe K *et al.* Flavour Hund's coupling, Chern gaps and charge diffusivity in moiré graphene. *Nature* 2021; **592**: 43–8.

5. Zhang Z, Xie J, Zhao W *et al.* Engineering correlated insulators in bilayer graphene with a remote Coulomb superlattice. *Nat Mater* 2024; **23**: 189–95.

6. Blöchl PE. Projector augmented-wave method. *Phys Rev B Condens Matter* 1994; **50**: 17953–79.

7. Kresse G, Furthmüller J. Efficiency of ab-initio total energy calculations for metals and semiconductors using a plane-wave basis set. *Comput Mater Sci* 1996; **6**: 15–50.

8. Anisimov VI, Zaanen J, Andersen OK. Band theory and Mott insulators: Hubbard U instead of Stoner I. *Phys Rev B* 1991; **44**: 943–54.

9. Grimme S, Antony J, Ehrlich S *et al.* A consistent and accurate ab initio parametrization of density functional dispersion correction (DFT-D) for the 94 elements H-Pu. *J Chem Phys* 2010; **132**: 154104.

10. Grimme S, Ehrlich S, Goerigk L. Effect of the damping function in dispersion corrected density functional theory. *J Comput Chem* 2011; **32**: 1456–65.

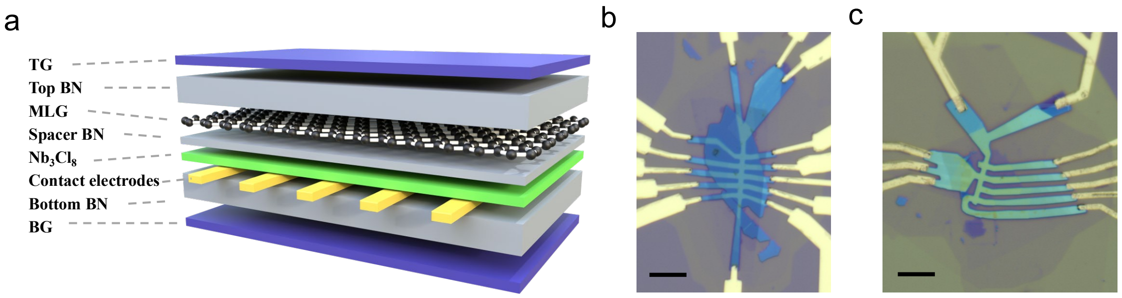

Fig. S1 Schematic diagram of MLG/hBN/Nb_3Cl_8 devices. (a) Schematic of dual-gated device structure. (b, c) Optical microscope image of the devices M1 (b) and M2 (c). Scale bar: 10μm.

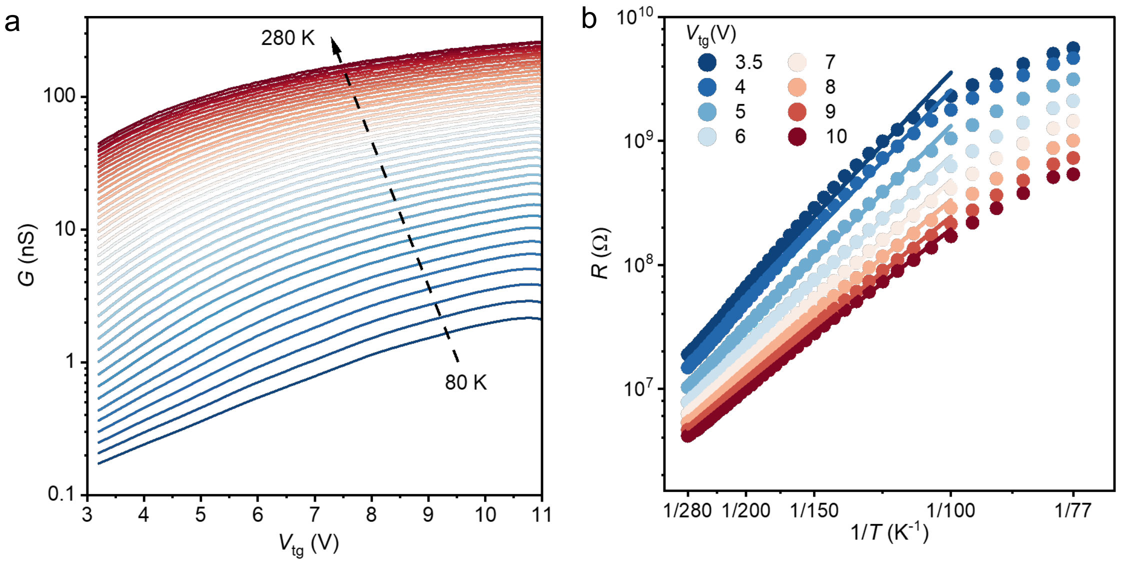

Fig. S2 Four-probe measurements of Device S1. (a) The four-terminal conductance of Nb_3Cl_8 as a function of top gate voltage V_{tg} from 80 K to 280 K with $V_{bg} = V_{tg}$. (b) The temperature-dependent four-terminal resistance at different gate voltages with the Arrhenius formula fitting. The extracted thermal activation gap is plotted in Fig. 1h of main text.

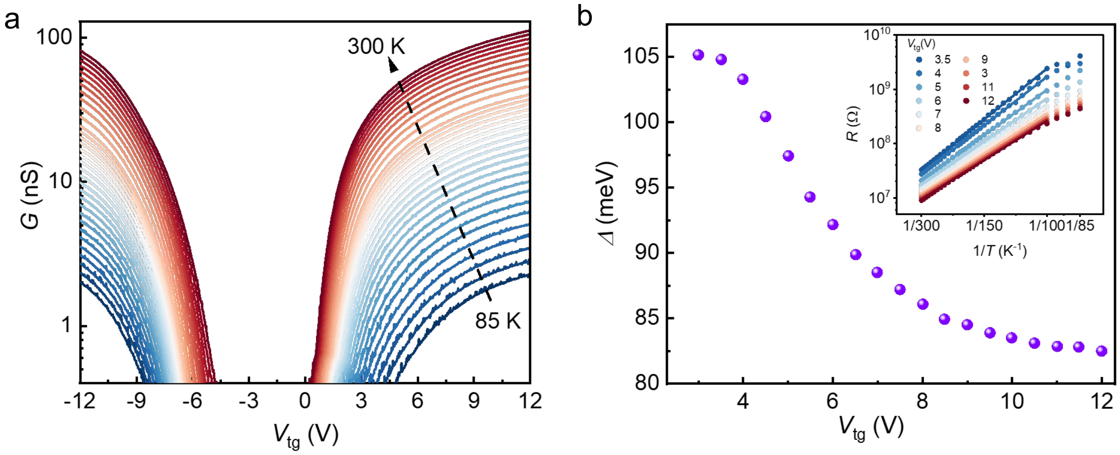

Fig. S3 Transport measurements on device S2. (a) The two-terminal conductance of Nb_3Cl_8 as a function of top gate voltage at different temperatures. (b) With increasing of the gate voltage, the thermal activation gap is decreased gradually, which is consistent with that observed in device S1 (Fig. 1h of main text). Insert: Solid lines represent Arrhenius fits to extract the activation gap.

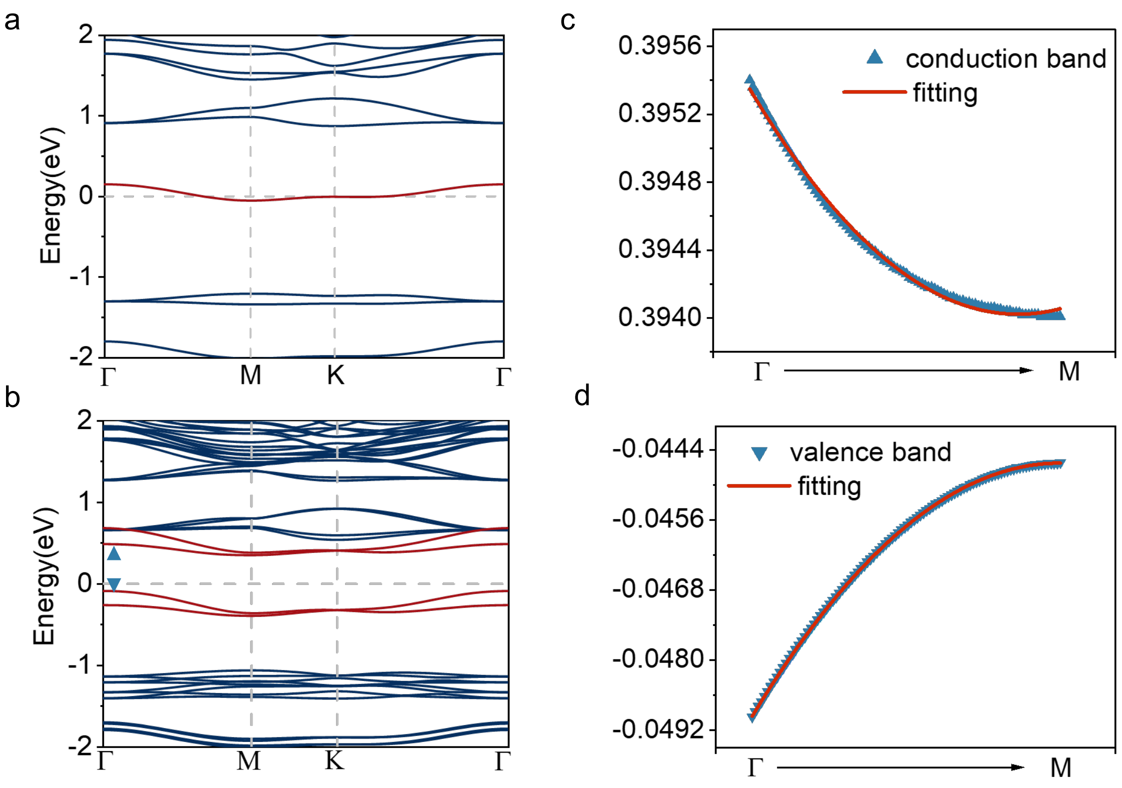

Fig. S4 DFT calculations of band structure and effective mass. (a) The band structure of monolayer Nb_3Cl_8, without considering the electron correlation effect. (b) The band structure of bulk Nb_3Cl_8 after considering the electron correlation effect with Hubbard term $U_{eff} = 2$ eV. (c, d) The energy bands near the bottom of the conduction band and the top of the valence band in (b), which are fitted with the quadratic coefficients B_2 of 24.62 and 60.36, respectively. All calculations were performed for $U_{eff} = 2$ eV.
